# Inequalities in mortality from leading cancers in districts of England from 2002 to 2019: population-based high-resolution spatiotemporal analysis of vital registration data

**DOI:** 10.1016/S1470-2045(23)00530-2

**Published:** 2023-12-11

**Authors:** Theo Rashid, James E Bennett, David C Muller, Amanda J Cross, Jonathan Pearson-Stuttard, Perviz Asaria, Hima lyathooray Daby, Daniela Fecht, Bethan Davies, Majid Ezzati

**Affiliations:** 1 Department of Epidemiology and Biostatistics, School of Public Health, Imperial College London, London, UK; 2 MRC Centre for Environment and Health, School of Public Health, Imperial College London, London, UK; 3 Cancer Screening and Prevention Research Group (CSPRG), Department of Surgery and Cancer, Imperial College London, London, UK; 4 Health Analytics, Lane Clark & Peacock LLP, London, UK; 5 Department of Cardiology, Imperial College NHS Trust, London, UK; 6 UK Small Area Health Statistics Unit, Imperial College London, London, UK; 7 Abdul Latif Jameel Institute for Disease and Emergency Analytics, Imperial College London, London, UK; 8 Regional Institute for Population Studies, University of Ghana, Accra, Ghana

## Abstract

**Background:**

Cancers are the leading cause of death in England. Our aim was to estimate trends from 2002-2019 in mortality from leading cancers for the 314 districts in England.

**Methods:**

We used vital registration data in England from 2002 to 2019 for ten leading cancers by sex according to the total number of deaths over the study period, and a residual group of all other cancers. We used a Bayesian hierarchical model to obtain robust estimates of age- and cause-specific death rates. We applied life tables to calculate the probability of dying between birth and 80 years of age by sex, cancer cause of death, district and year. We report Spearman rank correlation between the probability of dying from a cancer and district-level poverty in 2019.

**Findings:**

In 2019, the probability of dying from a cancer ranged from 0.10 (95% credible interval 0.10-0.11) to 0.17 (0.16-0.18) for women and from 0.12 (0.12-0.13) to 0.22 (0.21-0.23) for men. The most unequal cancers were lung cancer for women (3.7-times (3.2-4.4) variation between the districts with the highest and lowest probabilities of dying) and stomach cancer for men (3.2-times (2.6-4.1)). The cancers with the least geographical variability were lymphoma and multiple myeloma (1.2-times (1.1-1.4) for women and 1.2-times (1.0-1.4) for men), and leukaemia (1.1-times (1.0-1.4) for women and 1.2-times (1.0-1.5) for men). The correlation between probability of dying from a cancer and district poverty was 0.74 (0.72-0.76) for women and 0.79 (0.78-0.81) for men. The probability of dying declined in all districts from 2002 to 2019: the reductions ranged from 6.6% (0.3-13.1%) to 30.1% (25.6-34.5%) for women and 12.8% (7.1-18.8%) to 36.7% (32.2-41.2%) for men.

**Interpretation:**

Cancers with modifiable risk factors and potential for screening for pre-cancerous lesions had heterogeneous trends and the greatest inequality. Reducing these inequalities requires addressing factors affecting both incidence and survival at the local level.

## Introduction

Mortality from cancers in the UK and other industrialised countries has declined more slowly than from other major causes of death, and hence the share of deaths from cancers has steadily increased.^1^ Cancers are now the leading cause of death in England, overtaking cardiovascular diseases.^2^

Subnational data on trends in cancer mortality are currently limited to large areas.3,4 Small-area data can also guide primary prevention strategies to reduce incidence, and health care planning and delivery to improve survival. We report trends in cancer mortality for 314 districts in England from 2002 to 2019 and the association with poverty.

## Methods

### Data

We used vital registration data for all deaths in England from 2002 to 2019 (8,648,191 death records). We used postcode of residence at death registration to assign each death to a local authority district (referred to as district hereafter). England had 314 districts in 2020. Records were categorised into the following age groups: 0, 1-4, 5-9, 10-14, …, 80-84 and ≥85 years. We did not use 130 death records (<0.001%) for which sex was not recorded.

**Figure 1:**
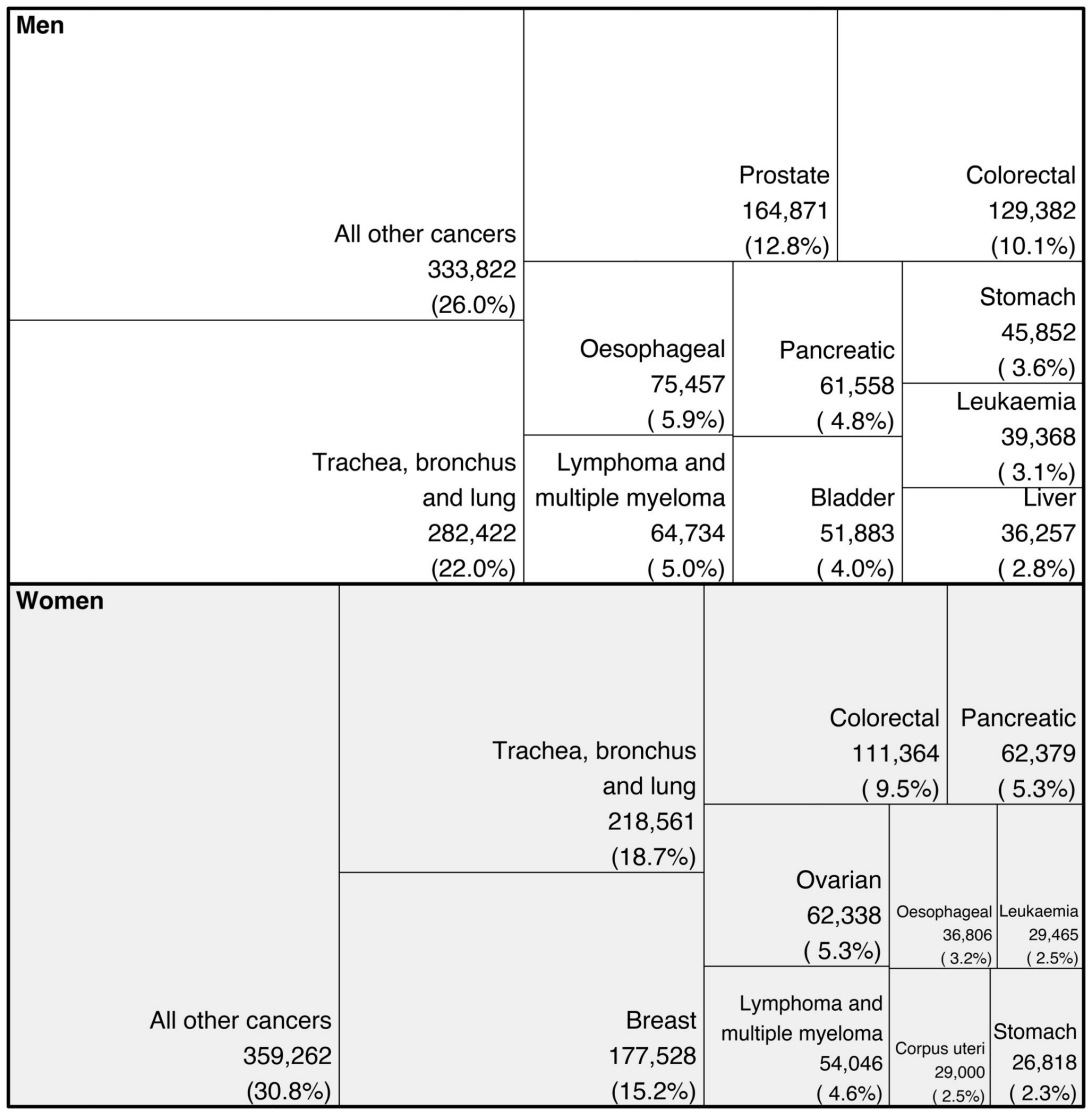
Total number of deaths for the ten leading cancers in England from 2002 to 2019 by sex. See the Appendix (p6)for ICD-10 codes in each category.

We used ICD-10 codes for underlying cause of death to assign each death to 136 cause groups of the WHO Global Health Estimates (GHE) study. We used the top ten leading cancer causes of death for each sex according to the total number of deaths from 2002 to 2019 (Figure 1) for cause-specific analysis, as well as a residual group comprising all other cancer deaths (Appendix p6).

Mid-year population data by age group, district, year, and sex were obtained from the ONS. We used data on income deprivation from the English Indices of Deprivation for 2019 (referred to as poverty hereafter), defined as the proportion of the district population claiming income-related benefits due to being out-of-work or having low earnings. The deprivation data are available at the Lower-layer Super Output Area (LSOA) level, which we aggregated to the district level by taking the population-weighted average. We did not use data on ethnicity because it is not recorded on death certificates and district-level data are only available for the 2011 census year.

### Statistical analysis

The number of deaths in each age group, district, year, and cancer cause group is small, which means that death rates calculated from observed data have an apparent variability from year to year, or from district to district, that is larger than the true differences in the risk of death. We adapted a Bayesian hierarchical model from previous studies^5,6^ to obtain stable estimates of death rates by sharing information across age groups, districts and years. In our hierarchical model, death rates for each age group, district and year were informed by data in that district-age-year unit as well as by those in adjacent age groups, adjacent years, and nearby districts. We did not use district-level socioeconomic variables like poverty as a covariate in the model because such data are only available for some years, and the methodology for their calculation may vary from year to year. Detailed model specification is presented in the Appendix (pp2–4 and p7).

We conducted all analyses separately by sex and cancer cause of death. In addition, we ran a model for all cancer deaths combined. The results for total cancer mortality using the combined model and the sum of cause-specific models were nearly identical (correlation coefficient across all years 0.99 for both sexes; mean absolute difference in probability of death <0.01 for both sexes).

Our primary reporting outcome is the unconditional probability of dying between birth and 80 years of age, which is the probability of death in the absence of competing causes of death, calculated by applying life tables to the posterior estimates of age-specific death rates (detailed calculation in the Appendix (p5)). We used probability of death because it has an intuitive interpretation, and because, unlike age-standardised death rate, it does not depend on the choice of standard population. We used unconditional probability of death because removing competing causes of death enhances comparability and equity.^7^ We limited the age range because the probability of death in the absence of competing causes equals 1.0 when the entire life course is considered. We selected 80 years of age as the upper bound because it covers a wide age range but does not include the very oldest ages where multimorbidity makes the assignment of cause of death increasingly difficult. We also calculated age-standardised death rates. The correlation coefficients between age-standardised death rates and the probability of dying between birth and 80 years of age ranged from 0.93 to >0.99 across sex-year-cancer combinations for the years 2002 and 2019 (Appendix pp8–10).

The reported 95% credible intervals (CrI) represent the 2.5^th^ to 97.5^th^ percentiles of the posterior distribution of estimated probability of death. We also report the posterior probability (PP) that the estimated change over time in a district represents an increase versus a decrease in the probability of dying between birth and 80 years of age. If the estimated probability of dying is the same in 2002 and 2019 and an increase is statistically indistinguishable from a decrease, there is a 0.5 PP of an increase and a 0.5 PP of a decrease. PPs more distant from 0.5 indicate more certainty. We report Spearman rank correlation between the district (rank of) probability of dying between birth and 80 years and (rank of) poverty in 2019, together with its 95% CrI.

### Role of the funding source

The funders of the study had no role in study design, data collection, data analysis, data interpretation, or writing of the report.

## Results

There were 2,453,173 deaths from cancers in England from 2002 to 2019; of these, 1,533,703 (62.5%) deaths occurred before 80 years of age. Of cancer deaths before 80 years of age, 697,953 (45.5%) were deaths in women and 835,750 (54.5%) in men. Nationally, the probability of dying from a cancer before 80 years of age declined for both sexes, from 0.16 to 0.13 for women and 0.22 to 0.17 for men from 2002-2019.

**Figure 2:**
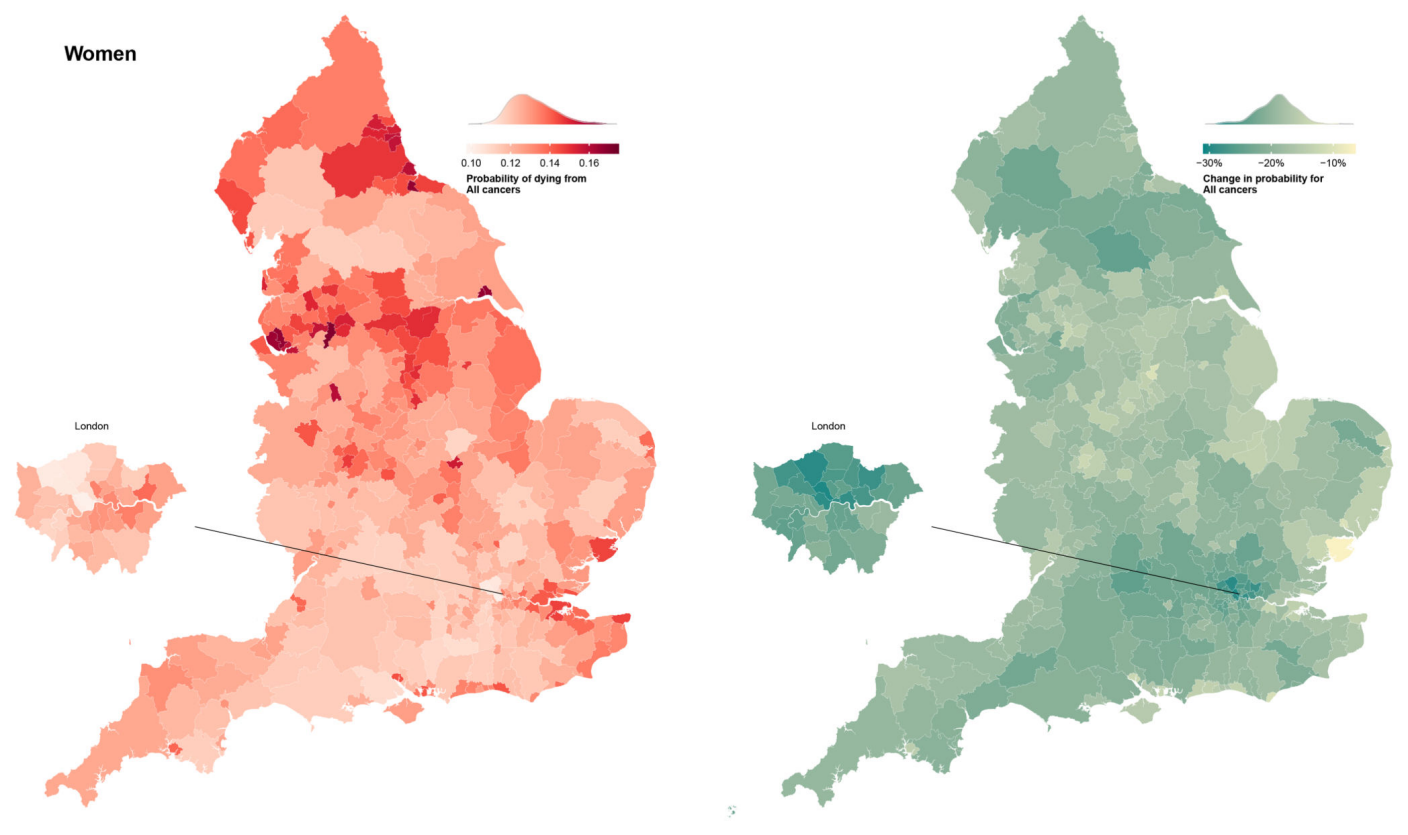
Probability of dying from a cancer between birth and 80 years of age in 314 local authority districts in England in 2019 and change from 2002 to 2019. See the Appendix (pp11–33)for results for specific cancers.

In 2019, the probability of dying from a cancer before 80 years of age ranged from one in ten (0.10 (95% CrI 0.10-0.11)) in Westminster to one in six (0.17 (0.16-0.18)) in Manchester for women, and from one in eight (0.12 (0.12-0.13)) in Harrow to one in five (0.22 (0.21-0.23)) in Manchester for men (Figure 2). The highest probabilities of dying were in northern cities such as Liverpool, Manchester, Hull and Newcastle, and in coastal areas to the east of London.

**Figure 3:**
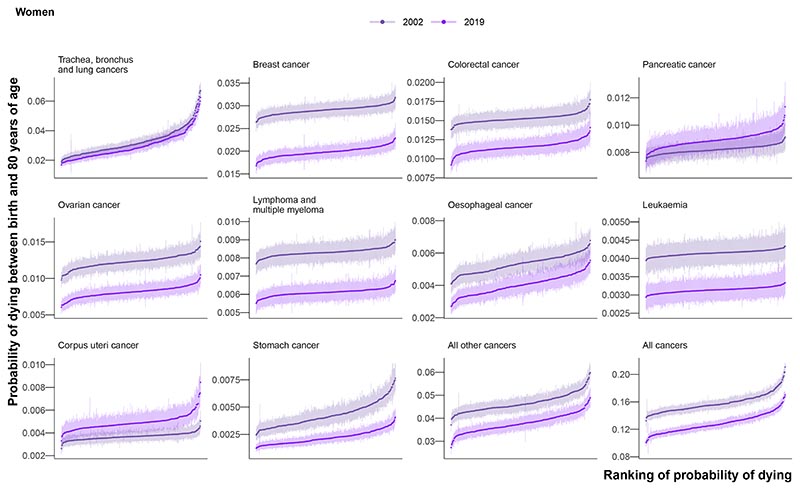
Ranked probability of dying between birth and 80 years of age in 314 local authority districts in England in 2002 and 2019 for the ten leading cancers. Each point shows one district and the vertical line going through the point its 95% credible interval. The districts were ranked by the posterior median estimate for the probability of dying.

Among the ten leading cancer causes of death (Figure 1), for the mean age at death in 2019 among those who died before 80 years of age varied from 63.0 years for breast cancer to 69.0 years for lung cancer in women and from 66.1 years for the combined category of all other cancers to 72.2 for prostate cancer in men. The leading cancer cause of death for both sexes was lung cancer, with 218,561 deaths (18.7% of all cancer deaths; 148,551 before 80 years) in women and 282,422 deaths (22.0%; 201,862) in men from 2002-2019 (Figure 1). Lung cancer was one of the most unequal cancers, with 3.7-times (3.2-4.4) and 3.0-times (2.7-3.5) variation across districts in 2019 in the probability of death for women and men, respectively. The highest probabilities of dying were in the urban North West and North East (Appendix pp11–33), with the highest probability of death in 2019 being 0.06 (0.06-0.07) in Knowsley for women and 0.07 (0.07-0.08) in Manchester for men. The distribution of district-level probabilities of dying from lung cancer was nearly identical in 2002 and 2019 for women, whereas for men the probabilities declined everywhere (Figure 3).

Lung cancer was followed by major sex-specific cancers – breast cancer (177,528 (15.2%) deaths) for women and prostate cancer (164,871 (12.8%)) for men. In 2019, deaths from these cancers had less district-level inequality than from lung cancer (Figure 3), although the probability of dying from prostate cancer was noticeably lower in northwest London than elsewhere (Appendix pp11–33). The probability of dying from ovarian cancer was also lower in London, in contrast to corpus uteri cancer, where east London had the highest probabilities (Appendix pp11–33). High probabilities of death from liver cancer, one of the leading causes of cancer mortality among men, were very concentrated in the North West, the North East, and central London. Stomach cancer mortality showed vast inequality (3.3-times (2.4-4.6) for women and 3.2-times (2.6-4.1) for men), with the highest probabilities of dying in the urban North West. The cancers with the least geographical variability were lymphoma and multiple myeloma (1.2-times (1.1-1.4) for women and 1.2-times (1.0-1.4) for men) and leukaemia (1.1-times (1.0-1.4) for women and 1.2-times (1.0-1.5) for men).

For women, the district-level probabilities of dying from stomach, oesophageal, lung and the residual category of all other cancers were correlated, with pairwise correlation coefficients ranging 0.51-0.75 (Appendix pp34–35). For men, the probability of dying from lung cancer was correlated with those of stomach, liver, bladder, colorectal, oesophageal, and all other cancers. There were particularly high correlations between the probabilities of dying from bladder and oesophageal cancers (0.71), and from oesophageal and colorectal cancers (0.69). Probabilities of dying from leukaemia, lymphoma and multiple myeloma, and pancreatic and corpus uteri cancers were only weakly correlated with probabilities for other categories.

**Figure 4:**
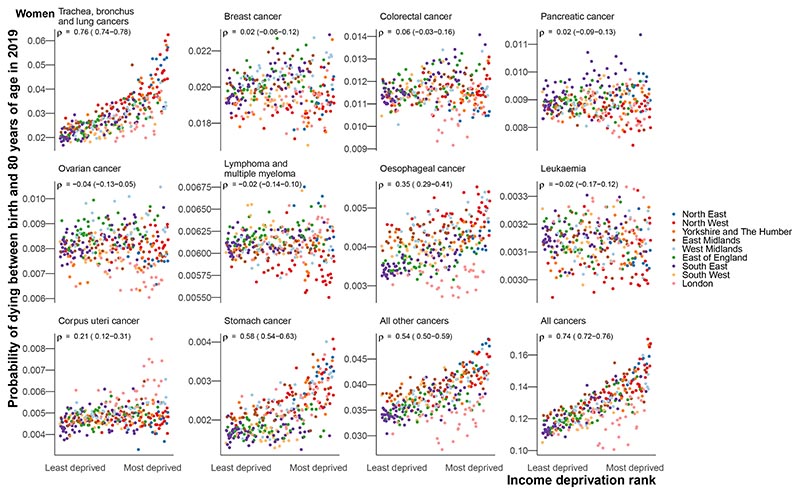
Local authority district probability of death in 2019 in relation to poverty for each of the leading cancers. The points are coloured by the regions in England. The median Spearman rank correlations (ρ) between the probability of dying from the cancer and poverty ranking and the corresponding 95% credible intervals, calculated at the posterior sample-level, are in the top left of each panel.

The probability of dying from a cancer was associated with poverty for both sexes, with Spearman correlation coefficients of 0.74 (0.72-0.76) for women and 0.79 (0.78-0.81) for men (Figure 4). In part, this was due to the probability of dying from the leading cancer for both sexes, lung cancer, exhibiting strong correlations with poverty (0.76 (0.74-0.78) for women and 0.85 (0.83-0.87) for men). There were also strong correlations between poverty and mortality from stomach cancer (0.58 (0.54-0.63) for women and 0.64 (0.60-0.67) for men), all other cancers (0.54 (0.50-0.59) and 0.56 (0.52-0.60)) and liver cancer (0.57 (0.51-0.62) for men for whom it was analysed). The probabilities of dying from breast cancer (0.02 (-0.06 to 0.12), prostate cancer (0.13 (0.05-0.21)), pancreatic cancer (0.02 (-0.09 to 0.13) and 0.01 (-0.11 to 0.14)), lymphoma and multiple myeloma (-0.02 (-0.14 to 0.10) and -0.05 (-0.16 to 0.07)), leukaemia (-0.02 (-0.17 to 0.12) and 0.09 (-0.04 to 0.22)), and colorectal cancer in women (0.06 (-0.03 to 0.16)) showed little or no association with poverty. Those in poor districts of London had lower probabilities of dying from lung, colorectal, oesophageal, bladder (men), and all other cancers than in comparably poor districts in the rest of the country (Figure 4).

**Figure 5:**
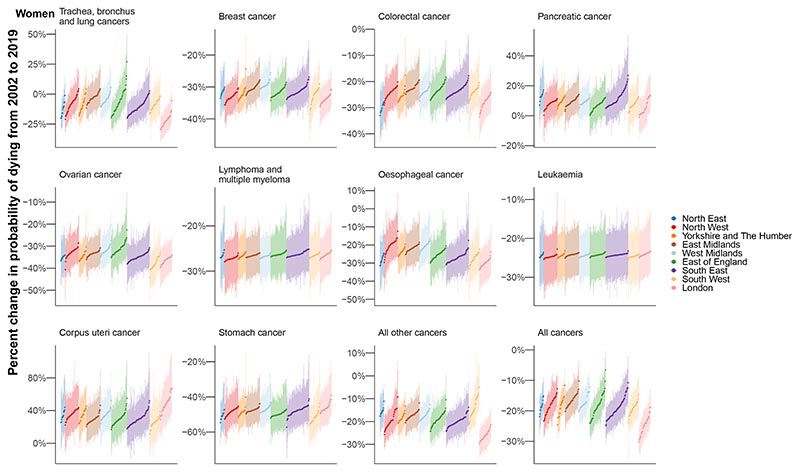
Change in probability of dying between birth and 80 years of age in 314 local authority districts in England in 2002 and 2019 for the ten leading cancers. Each point shows one district and the vertical line going through the point its 95% credible interval. The districts were first ordered by region and then ranked by the posterior median estimate for change in the probability of dying. The points are coloured by the regions in England.

The probability of dying from a cancer before 80 years of age declined from 2002-2019 in every district for both sexes with a PP >0.97, with median reduction across districts of 18.7% for women and 23.4% for men. However, these reductions occurred at varying rates (Figure 5). For women, the greatest district-level reduction in the probability of dying from a cancer (30.1% (25.6-34.5%)) was nearly five times that of the smallest (6.6% (0.3-13.1%)). For men, the largest decrease was triple that of the smallest, 36.7% (32.2-41.2%) compared to 12.8% (7.1-18.8%). Districts in London achieved the largest declines.

Among cause categories, the largest reductions in mortality were for stomach cancer, with the declines from 2002-2019 in probability of death ranging 39.1-57.2% across districts for women, and 51.5-58.8% for men (PP >0.99). The probability of dying from oesophageal cancer also decreased in every district for women, but varied for men from a 42.9% (27.7-56.0%) decrease in Plymouth to a 7.0% (-19.8% to 42.4%) increase in Gosport (Figure 5and Appendix pp11–33).

Lung cancer mortality decreased everywhere for men with PP >0.99, but for women, there were mixed trends. The largest declines were in London, (largest decrease: 29.5% (18.5-38.8%) in Newham), whereas the probability of dying increased in many districts in the East of England, (largest increase: 27.0% (6.8-49.7%) in Tendring) (Figure 5and Appendix pp11–33). The PP that the observed decline for lung cancer in women was a true decline was >0.80 in 197 (62.7%) districts. Women in 4 (1.3%) districts experienced an increase in lung cancer mortality with a PP >0.80, and in the remaining 113 (36.0%) districts there was no detectable change at this PP.

The probability of dying from pancreatic cancer increased in all but one sex-district combination, but the PP of the observed increase was a true increase was >0.80 in 259 (82.5%) districts for women and 302 (96.2%) districts for men. The median increase across districts was 8.6% for women and 6.7% for men, and the largest increases were in the South East (Figure 5). Liver cancer for men (median increase 78.3%) saw increases in mortality in all districts with PP >0.98, and corpus uteri cancer for women (median increase 31.7%) saw increases in mortality in all but one district with PP>0.80. There were increases in male liver cancer mortality in excess of 100% in 32 (10.2%) districts, mostly in northern England (Figure 5). All districts that experienced an increase in pancreatic cancer with a PP >0.80 also experienced an increase in a second cancer at the same PP (corpus uteri for women, liver for men). Women in 3 (1.0%) districts experienced an increase in three cancers (lung, pancreatic and corpus uteri cancer) with a PP >0.80.

## Discussion

Although overall cancer mortality decreased everywhere, the gains were unequal, with the largest declines almost five times that of the smallest. Cancers with strong links to behavioural and environmental risk factors, and those with screening for pre-cancerous lesions^8^ (from the cancer groups in this study: lung, colorectal, breast, oesophageal, stomach, liver, and bladder cancers), saw some of the largest inequalities across districts and the widest variation in how much they changed. The cancers showing the least inequality, lymphoma and multiple myeloma and leukaemia, were those with weaker and more heterogeneous links to modifiable risk factors.

A strength of our study is the presentation of mortality from leading cancers, as well as all cancers together, for subnational spatial units in England over a period of substantial change in public health programmes and healthcare technology and provision. We used a Bayesian hierarchical model to robustly estimate death rates for different cancers, together with uncertainty in these areas, by sharing information over age, space, and time. A limitation of our work is that we only analysed deaths from the ten leading causes of death from cancer and did not split the residual group into more specific cancer groups. This was done so that there were sufficient deaths in each cause group for robust inference of age-specific death rates at the district level. Each cancer group was analysed separately rather than in a single model, which should be explored in methodological work. Our reporting metric, the probability of dying between birth and 80 years of age, intuitively presents mortality over the life-course, but does not include differences in age groups beyond its upper age limit. We found that this metric was highly correlated with age-standardised death rates. Although districts are valuable units for analysis as they are used for resource allocation and policy implementation, previous studies on total mortality have found heterogeneity among units smaller than districts.^5,6^ Furthermore, we did not analyse or present data by healthcare geographies because these do not map to districts, and because regional cancer services vary within England. For example, there may be a single regional cancer centre in some areas, or a single specialist centre for a particular malignancy. There are also factors including patient choice (of where to receive care), age (younger patients may be treated at specialist children’s hospitals), cancer stage (late stage cancers are more aggressive and might be treated in a specialist centre), and year (health geographies are subject to change whenever the NHS undergoes a reorganisation) which mean people in the same district may not be treated at the same hospital for each cancer. The population in each district can change because of migration, both within the country and from overseas. Therefore, changes in cancer mortality should not be entirely attributed to changes in the health of the population, although studies from both the UK^5^ and USA^9^ have shown that migration alone is unlikely to fully explain trends in health outcomes. Moreover, the majority of moves in the UK are within the same district,^10^ which does not affect the change in mortality in a given district. The correlations with poverty were reported at the district level, whereas there are variations in both mortality and poverty within each district.^5,6^ The correlations between cancer risk and individual or household poverty may be even stronger because aggregation tends to attenuate correlation. To ensure the underlying cause of death data are comparable between places and times, the UK has national guidelines for death certification, as well as algorithms for the computer-based assignment of the underlying cause of death.^11^ For example, a validation study found that that death certificates in the UK accurately identified deaths from prostate cancer.^12^ Despite this, there might be differences in the cancer cause of death assignment between districts.

Cancer mortality depends on both incidence and survival. Incidence is influenced by three factors: exposure to risk factors for cancer, including smoking, alcohol use, obesity, infections, and occupational and environmental exposures; the uptake of preventative treatments such as vaccinations; and the implementation of screening for pre-cancerous lesions. In England, smoking, alcohol use and obesity are higher where cancer mortality is highest, generally in the North.^13–15^ The rise and fall in female smoking have lagged behind men by about 20-30 years,^16^ leading to the mix of increase and decrease in female lung cancer mortality while that of males declined everywhere. Similarly, after peaking in the mid-2000s, alcohol consumption has declined, and is lowest in London.^13,17^ In contrast, obesity has risen,^15^ mirrored by a rise in diabetes.^15^ These trends could be partly responsible for cancers associated with diabetes and obesity increasingly co-occurring with those due to smoking and alcohol use, especially in men among whom smoking rates declined earlier. We observed substantial increases in liver cancer mortality for men, especially in northern districts, which mirror a long-run trend in the USA where it has increased both nationally and in nearly all counties.^18^,^19^ The observed trends in liver cancer mortality could be linked to the heterogeneous trends in its major risk factors, namely infections, smoking, alcohol use and diabetes.^20–23^ Differences between men and women in the temporal and geographical distribution of tobacco smoking, alcohol use, poor diet, and obesity may potentially account for our observation that oesophageal cancer mortality decreased in all districts for women, but increased in some districts for men. Stomach cancer exhibited the largest decreases in mortality, as well as substantial inequality in the level across districts. In part this may be due to variable prevalence of, and heterogeneous decrease in, *H pylori* infection.

Survival is influenced by awareness and utilisation of care, access and barriers to screening and early diagnosis, tumour factors (stage, topography, histology), comorbidities, and the quality of care. The UK has performed badly in terms of survival compared to other European countries.^24^ There were also subnational variations in survival for a number of cancers, both for cancers with relatively high average survival (e.g., colorectal and corpus uteri cancers) and those with relatively low average survival (e.g., pancreatic and liver cancers).^3^ This survival variation, together with variations in incidence, would drive the observed variation in mortality.

Combinations of risk factors and healthcare can also explain London’s better performance for some cancers. Firstly, there can be differences in cancer risk factors between London’s ethnically diverse population and the rest of England. For example, smoking rates amongst Asian (8.3% of adults) and black (9.7%) groups are lower than the white ethnic group (14.4%).^25^ Secondly, there could be differences in the quality of healthcare, either because advanced treatments such as immunotherapies are more available, or, because patients can travel to a specialist hospital rather than their local hospital thanks to the higher density of hospitals. However, districts in East London had the highest mortality for corpus uteri cancer, which has higher incidence rates in the black ethnic group in England.^26^

Since the turn of the century, two major national policy initiatives in the UK have aimed to improve cancer care and survival. Firstly, the NHS Cancer Plan of 2000 targeted socioeconomic inequalities in cancer survival through an increase in expenditure, a focus on centralisation and specialisation of cancer services, and a greater use of multidisciplinary teams. Secondly, the National Awareness and Early Diagnostic Initiative, launched in 2008, addressed both patient factors, such as reducing the stigma around cancer and the barriers to seeing a doctor, and tumour factors, such as improving access to screening and optimising referral pathways. However, no consistent evidence was found of a direct impact on one-year survival after these successive initiatives.^27^,^28^ Recent NHS plans have renewed focus on survival targets and early diagnosis,^29^ as well as the roll out of a national lung cancer screening programme targeted at those with a history of smoking.^30^ Technological developments, including multicancer detection assays, cancer vaccines, new and emerging drugs, combination therapy, and a shift towards personalised cancer care as the cost of profiling tumours falls, all offer scope for advancement. However, equitable availability of novel diagnostics and treatments to all patients may lag behind successful development and approvals, and patients without access will be more likely to present with advanced cancers and receive less advanced treatment. Our results on heterogeneous trends in mortality, particularly for cancers with modifiable risk factors and potential for screening for pre-cancerous lesions, indicate that reducing these mortality inequalities requires addressing factors affecting both incidence and survival at the local level.

## Supplementary Material

Appendix

## Data Availability

The Small Area Health Statistics Unit does not release data to third parties except in the form of non-disclosive statistical tables or conclusions suitable for publication. Individual-record mortality data can be requested through the Office for National Statistics (ONS) (https://www.ons.gov.uk). Mid-year population estimates (https://www.ons.gov.uk/peoplepopulationandcommunity/populationandmigration/populationestimates/datasets/middlesuperoutputareamidyearpopulationestimates) and the English Indices of Deprivation 2019 (https://www.gov.uk/government/statistics/english-indices-of-deprivation-2019) are available online. The code for the model (https://globalenvhealth.org/code-data-download/) is also available online.

## Research in context

### Evidence before the study

We searched PubMed for articles published from database inception up to July 5, 2023, using search terms “mortality” AND “cancer” AND (“sub-national” OR “small area” OR “local”) AND (“trend” OR “time”) AND (“England” OR “United Kingdom”) for papers that had analysed trends in cancer mortality in England, with no language restrictions. We also searched for relevant reports through the websites of the Office for National Statistics and Public Health England. We found estimates of cancer mortality for health boards, which are much larger than districts. One study reported risk relative to the national population for multiple cancers in census wards, which is a finer resolution than district level, by aggregating data over a 25-year period but did not analyse change over time. We found two studies which investigated trends in cancer mortality: one looked at upper-tier local authorities from 1990 to 2016, of which there are fewer than half the number of districts in this study. The other aggregated communities into deciles of deprivation within each region and only focussed on four leading cancers from 2001 to 2016. None of the studies estimated cancer mortality for areas as small as districts nor included all leading cancer causes of death.

Elsewhere, cancer atlases have reported cancer mortality or incidence for coarse geographical units and aggregated multiple years of data rather than presenting information on trends. One study in the USA looked at trends in cancer mortality at the county level.

### Added value of this study

To our knowledge, we present estimates for trends in cancer mortality for districts in England at a higher resolution than previous studies. By using a Bayesian spatiotemporal model based on patterns of mortality over age, space and time, we obtained robust yearly estimates of cancer mortality for small geographies, together with uncertainty in these estimates.

### Implications of all the available evidence

Declines in overall cancer mortality have been unequal both geographically and among groups of cancers. The greatest geographical inequality was seen for cancers with modifiable risk factors and potential for screening for pre-cancerous lesions. Addressing risk factors like smoking and alcohol use, expanding access to and utilisation of screening for prevention and early detection, and improving the quality of care should all be used to reduce deaths where they remain highest. High-resolution spatiotemporal data can help identify where intervention is required and track progress.
