# Supplementary material for: Inequalities in mortality from leading cancers in districts of England from 2002 to 2019: population-based high-resolution spatiotemporal analysis of vital registration data
Source: Lancet Oncol. Author manuscript; Available in PMC 2024 Jan 12. (PMC7615518; doi:10.1016/S1470-2045(23)00530-2)
Supplement: Appendix [file EMS189489-supplement-Appendix.pdf]

Table of contents

Appendix Text 1: Specification of the Bayesian statistical model 2

Appendix Text 2: Calculation of the probability of dying between birth and 80 years of age................ 5

Appendix Table 1: Groups of underlying causes of death used in the analysis with ICD-10 codes..... 6

Appendix Table 2: Prior specification and dimensionality of the parameters in the Bayesian statistical model.. 7

Appendix Figure 1: Comparison of the probability of dying between birth and 80 years of age and the age-standardised death rate for site-specific cancers in 2002 and 2019.. 8

Appendix Figure 2: Maps of probability of death in 2019 and change in probability of death between 2002-19 for each of the leading cancers..11

Appendix Figure 3: Pairwise correlations between the leading cancers in 2019, by sex.....................34

References 36

Appendix Text 1: Specification of the Bayesian statistical model

We used a Bayesian hierarchical model to obtain robust estimates of death rates by age group, district (spatial unit) and year, which were then used to calculate the unconditional probability of dying between birth and 80 years of age. The model was run separately for each sex and cause group. The model was formulated to incorporate important features of death rates in relation to age, space and time. Deaths were divided into 19 age groups: 0, 1-4, 5-9, 10-14, ..., 80-84 and ≥85 years. The number of deaths in age group $a\ (= 1,\dots,19)$, district $s\ (= 1,\dots,314)$ and year $t\ (= 1,\dots,18)$ follows a binomial distribution

$$Deaths_{ast} \sim \text{Binomial}(m_{ast}, Population_{ast})\ .$$

Logit-transformed death rates, m_{ast}, were modelled as a function of time, age group and district, as

$$\text{logit}(m_{ast}) = \alpha_0 + \beta_0 t + \alpha_{1s} + \beta_{1s} t + \alpha_{2a} + \beta_{2a} t + \xi_{as} + \gamma_{at}\ ,$$

where α_0 is the overall intercept across all age groups and districts. β_0 quantifies the overall trend (over time) across all age groups and districts. α_{1s} and β_{1s} measure deviation from the overall intercept and trend terms, respectively, for each district, so that each district can have a different intercept and slope.

α_{2a} and β_{2a} measure deviation from the global level and trend, respectively, for each age group, so that each age group can have a different intercept and slope. We used first-order random walk priors on α_{2a} and β_{2a} to ensure similarity between adjacent age groups, with the form $A_a \sim \mathcal{N}(A_{a-1}, \sigma_A^2)$ for both age-specific terms α_{2a} and β_{2a}. This specification also avoids implausible age patterns of mortality that could occur if each age group were analysed separately. We constrained $\alpha_{21} = 0$ and $\beta_{21} = 0$ so each random walk was identifiable and centred on the corresponding overall term.

The spatial effects α_{1s} and β_{1s} were assigned spatially-structured intrinsic conditional autoregressive (ICAR) priors, allowing information to be shared locally between neighbouring

districts. The ICAR model requires all spatial units to have neighbours. Thus, the districts containing the Isle of Wight and the Isles of Scilly were each joined to the nearest mainland district based on road or ferry connections.

ξ_{as} is an age group-district interaction term, which quantifies district-specific deviations from the overall age group structure given by α_{2a}. This allows different districts to have different age-specific mortality patterns, and equivalently each age group's death rate to have a different spatial pattern. This interaction term was modelled as $\mathcal{N}(0, \sigma_{\xi}^2)$.

γ_{at} are first-order random walks over time that allow age group-specific non-linearity in the time trends. For each age group, they were modelled via similar first-order random walk priors to those above with $\gamma_{a1} = 0$ so that the terms were identifiable.

The model is adapted from previous studies[1,2] with the following differences:

- Spatial effects were modelled using ICAR priors rather than a nested hierarchy of random effects. Previous papers used a three-tier hierarchy with spatial units nested inside districts. This allowed units that fell in different districts to differ more than those within the same district, reflecting the relevance of district as a unit of resource allocation. Regions have no such policy implications and hence we implemented a fully spatial model. We also tested but did not prefer Besag York Mollié (BYM) spatial priors[3] because model fit did not improve compared with the ICAR model.
- We used a binomial likelihood instead of a negative binomial or beta-binomial likelihood. A beta-binomial was trialled but did not improve model fit. We also tested but did not include a random walk for each spatial unit because it did not improve model fit despite adding a lot of complexity.

Appendix Table 1 shows all model parameters, their priors and dimensions.

Hyperpriors

As in earlier analyses, weakly informative priors were used so that inference on the parameters was driven by the data.[1,4,5] All variance parameters of the random effects had $\sigma \sim \mathcal{N}^{+}(1)$ priors. For the global intercept and slope, we used $\mathcal{N}(0, 10)$.

Implementation

Inference was performed using Markov chain Monte Carlo in NumPyro (v0.10.0).[6] Where possible, non-centred parameterisations were used in the model to improve the efficiency of the NUTS samplers. We monitored convergence using trace plots and the R-hat diagnostic,[7] and thinned post burn-in samples to reduce memory use and computation time. We ran four chains with a warmup of 2000 iterations and then 10,000 further iterations and thinned the remainder by 40 to obtain 1,000 post-burn-in draws from the posterior distribution of model parameters.

The code for the model is available from http://globalenvhealth.org/code-data-download/.

Appendix Text 2: Calculation of the probability of dying between birth and 80 years of age.

The calculation of the probability of dying uses standard life table methods.[8] Life tables can be constructed using discrete age bands starting at age x and ending at age $x+n$. The inputs are the age-specific death rates, denoted as m_x, and the average person-years lived in the interval by those dying in the interval, denoted as ${}_na_x$, which is assumed here to be the midpoint of the age interval except in the oldest ages (e.g., above 80 or 85 years of age).

A lifetable starts with a hypothetical cohort and sequentially calculates the probability of dying in each age group,

$${}_nq_x = \frac{n \cdot {}_n m_x}{1 + (n - {}_na_x){}_nm_x}.$$

The probability of dying between birth and 80 is then calculated as

$${}_{80}q_0 = 1 - \prod_{x=0}^{80} (1 - {}_nq_x)\,.$$

Appendix Table 1: Groups of underlying causes of death used in the analysis with ICD-10 codes.*

Cancer	ICD-10 codes
Trachea, bronchus and lung cancers†	C33-C34
Breast cancer	C50
Prostate cancer	C61
Colorectal cancer	C18-C21
Pancreatic cancer	C25
Ovarian cancer	C56
Lymphoma and multiple myeloma	C81-C90, C96
Oesophageal cancer	C15
Bladder cancer	C67
Leukaemia	C91-C95
Corpus uteri cancer	C54-C55
Stomach cancer	C16
Liver cancer	C22
All other cancers ‡§	C00-C14, C17, C23-C24, C26-C32, C37-C39, C40-C41, C43-C44, C45-C49, C51-C53, C57-C60, C62-C63, C64-C66, C68, C69-C80, C97, D00-D48

* For neonates, who are not assigned an underlying cause of death, we used the ICD-10 code in the first position on the death record.
† Referred to as lung cancer in the text for brevity.
‡ The residual group also contained deaths from the "ill-defined diseases" GHE group (R00-R94, R96-R99, U07, U99), which were proportionately assigned between the residual groups for cancers, non-communicable diseases, cardiovascular diseases, and infections as part of a wider study on causes of death in England. The residual group also includes breast cancer for men. Bladder cancer and liver cancer were not leading cancers for women, so they are included in the residual group.
§ The next leading specific cancer sites in the residual group of all other cancers were bladder cancer, brain and nervous system cancers, and liver cancer for women, and brain and nervous system cancers, mesothelioma, and melanoma and other skin cancers for men.

Appendix Table 2: Prior specification and dimensionality of the parameters in the Bayesian statistical model. Subscripts are as follows: s – district; a – age group; t – year.

**Parameter name**	**Symbol**	**Prior**	**Dimension**
Overall intercept	α_0	$\mathcal{N}(0, 10)$	1
Overall slope	β_0	$\mathcal{N}(0, 10)$	1
District intercept	α_{1s}	ICAR	314
District intercept standard deviation	$\sigma_{\alpha_{1s}}$	$\mathcal{N}^+(1)$	1
District slope	β_{1s}	ICAR	314
District slope standard deviation	$\sigma_{\beta_{1s}}$	$\mathcal{N}^+(1)$	1
Age group intercept	α_{2a}	$\mathcal{N}\left(\alpha_{2,a-1}, {\sigma_{\alpha_{2a}}}^2\right)$	18
Age group intercept standard deviation	$\sigma_{\alpha_{2a}}$	$\mathcal{N}^+(1)$	1
Age group slope	β_{2a}	$\mathcal{N}\left(\beta_{2,a-1}, {\sigma_{\beta_{2a}}}^2\right)$	18
Age group slope standard deviation	$\sigma_{\beta_{2a}}$	$\mathcal{N}^+(1)$	1
Age group district interaction	ξ_{as}	$\mathcal{N}\left(0, \sigma_\xi^2\right)$	19 x 314
Age group district interaction standard deviation	σ_ξ	$\mathcal{N}^+(1)$	1
Age group random walk over time	γ_{at}	$\mathcal{N}\left(\gamma_{a,t-1}, \sigma_\gamma^2\right)$	19 x 17
Age group random walk over time standard deviation	σ_γ	$\mathcal{N}^+(1)$	1

Appendix Figure 1: Comparison of the probability of dying between birth and 80 years of age and the age-standardised death rate for site-specific cancers in 2002 and 2019. The age-standardised death rates were calculated using the population in England from 2011 for both sexes combined, which is a census year and the mid-point of our analysis period, as our standard population.

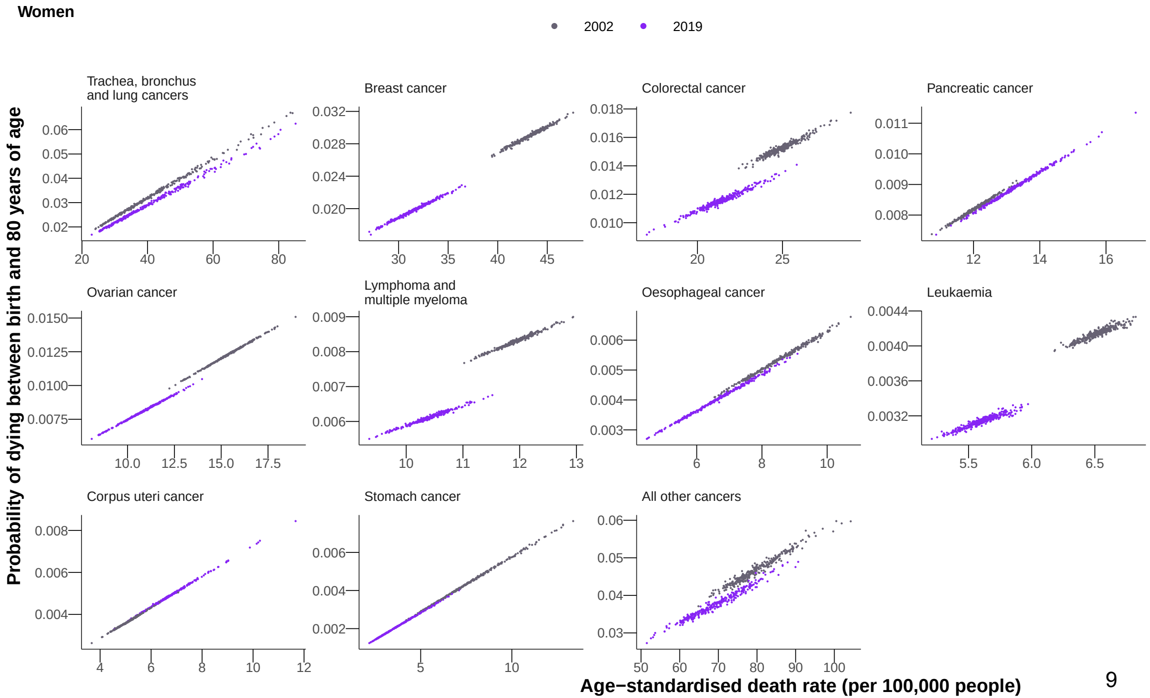
Women
2002
2019
Trachea, bronchus and lung cancers
Breast cancer
Colorectal cancer
Pancreatic cancer
Ovarian cancer
Lymphoma and multiple myeloma
Oesophageal cancer
Leukaemia
Corpus uteri cancer
Stomach cancer
All other cancers
Probability of dying between birth and 80 years of age
Age−standardised death rate (per 100,000 people)

Men

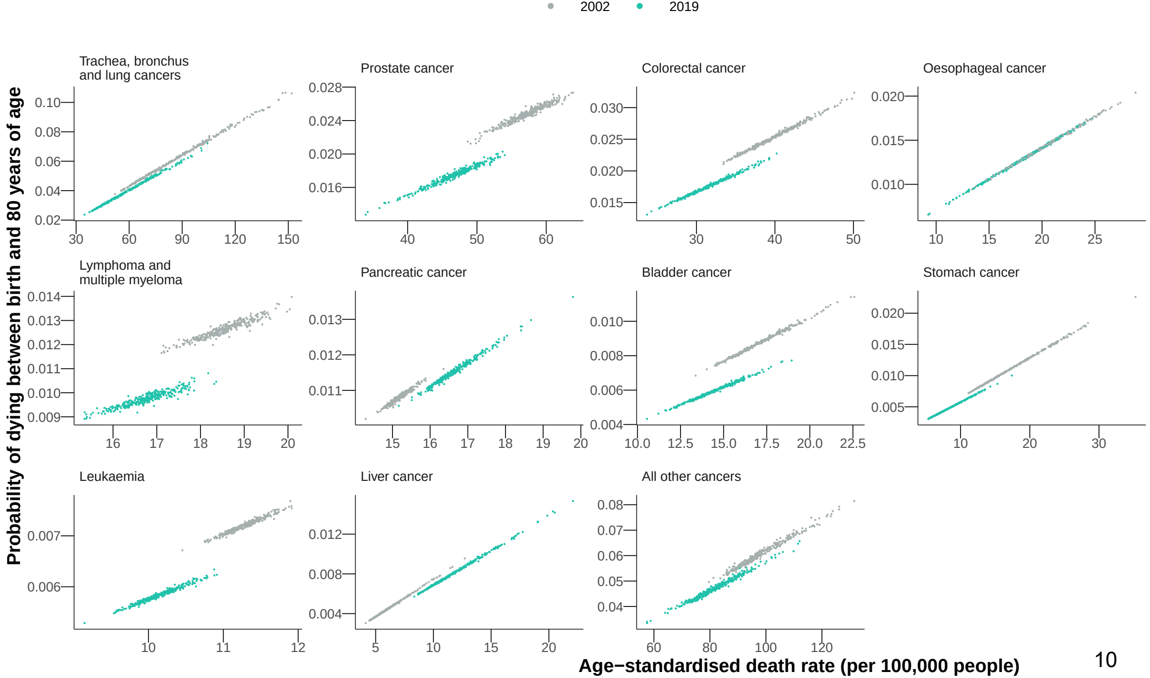

2002
2019
Trachea, bronchus and lung cancers
Prostate cancer
Colorectal cancer
Oesophageal cancer
Lymphoma and multiple myeloma
Pancreatic cancer
Bladder cancer
Stomach cancer
Leukaemia
Liver cancer
All other cancers
Probability of dying between birth and 80 years of age
Age−standardised death rate (per 100,000 people)

Appendix Figure 2: Maps of probability of death in 2019 and change in probability of death between 2002-19 for each of the leading cancers.

Trachea, bronchus and lung cancers, women

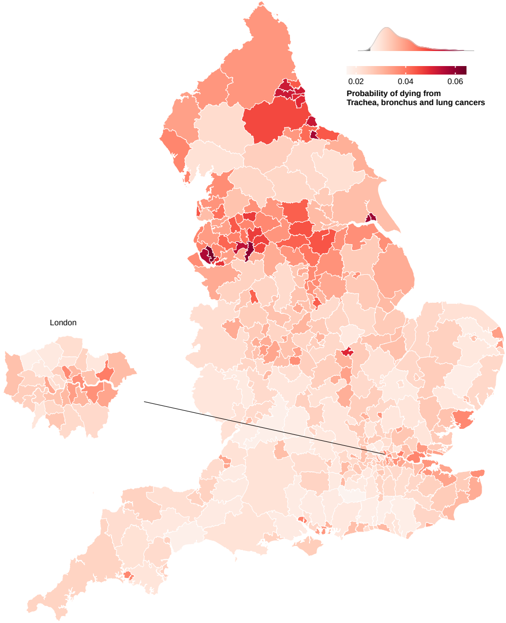

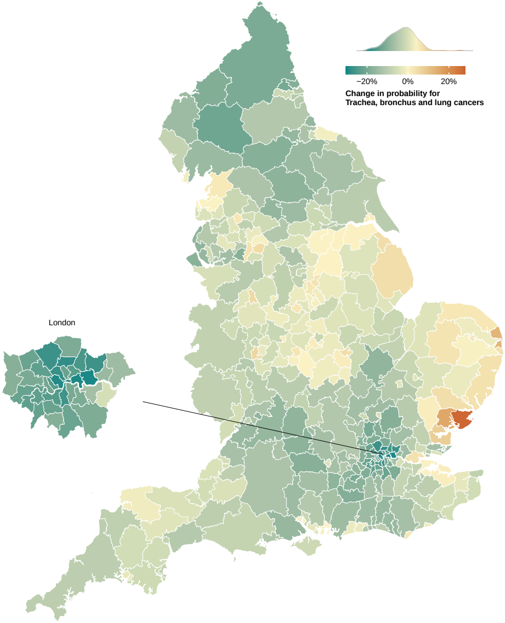

Trachea, bronchus and lung cancers, men

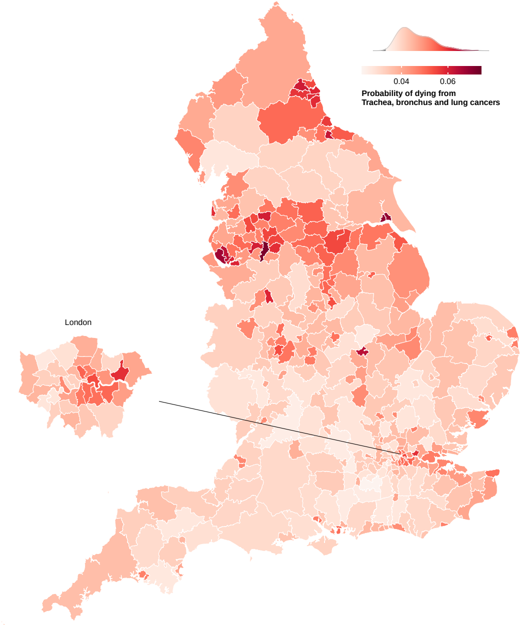

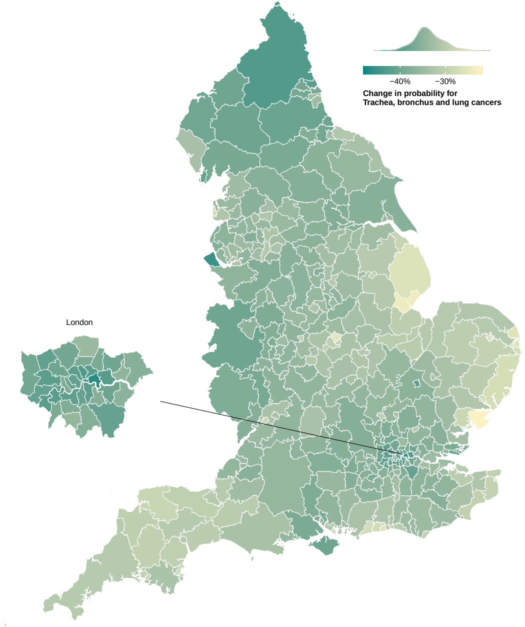

Colorectal cancer, women

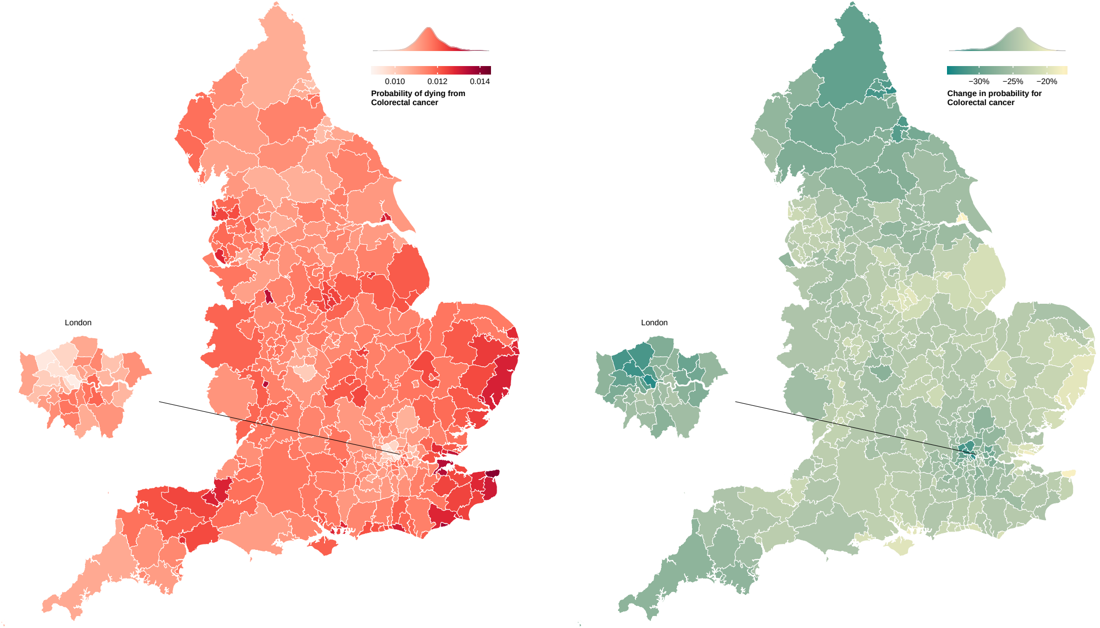

Colorectal cancer, men

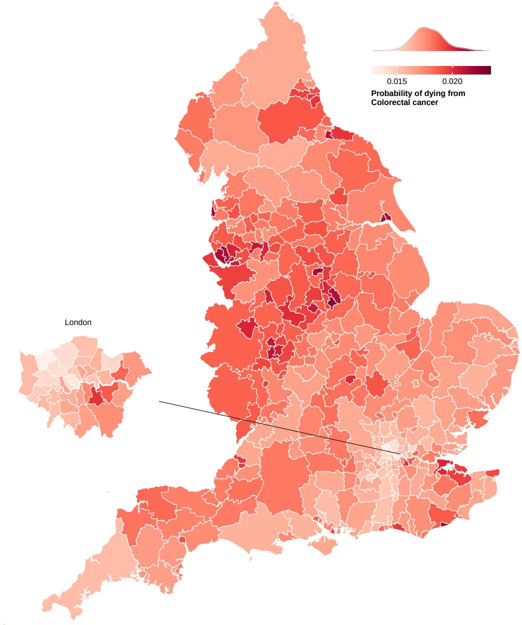

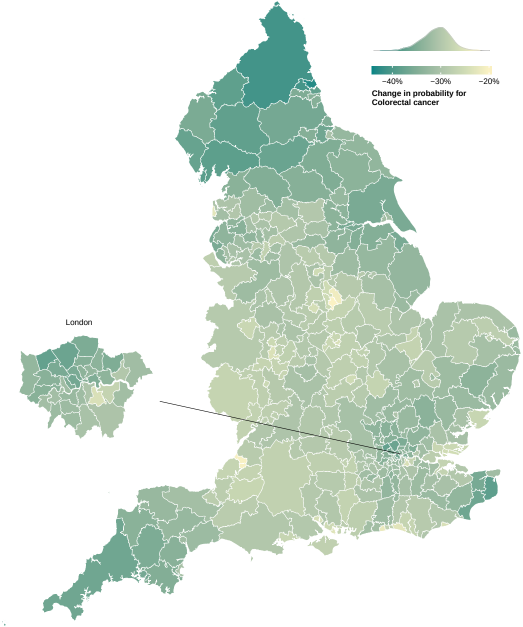

Oesophageal cancer, women

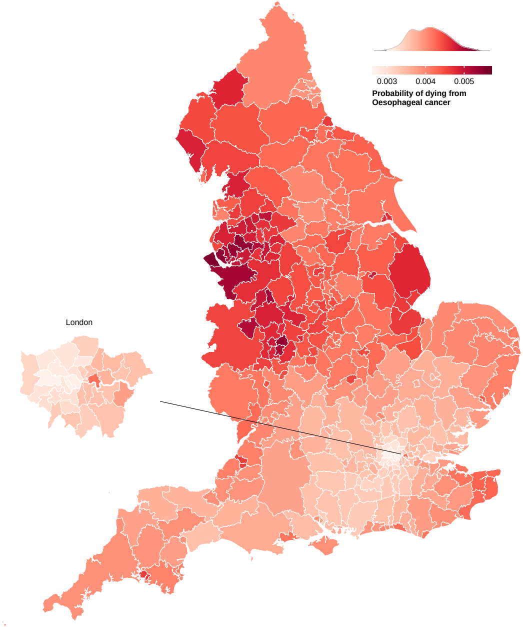

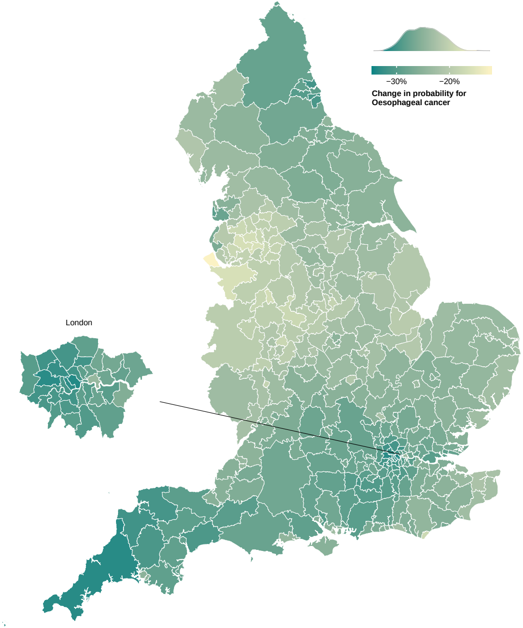

Oesophageal cancer, men

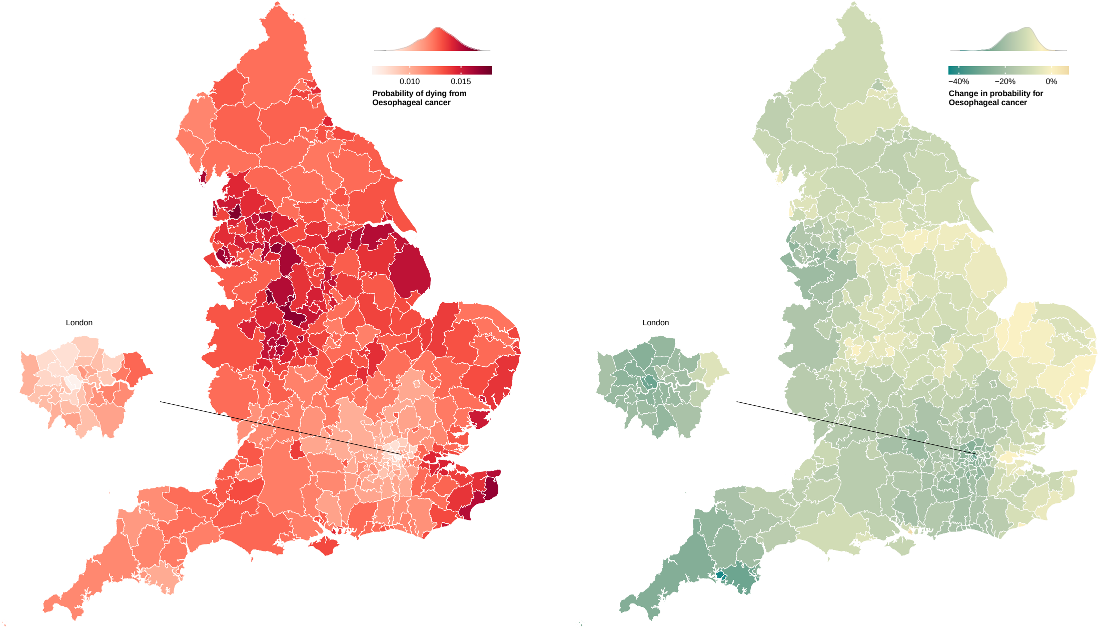

Oesophageal cancer, women

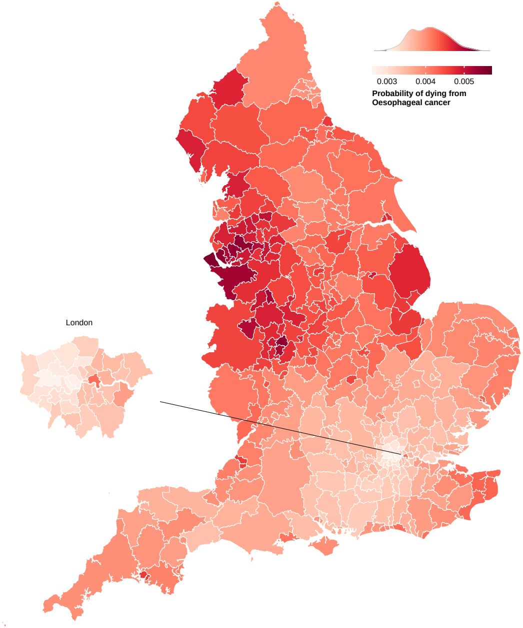

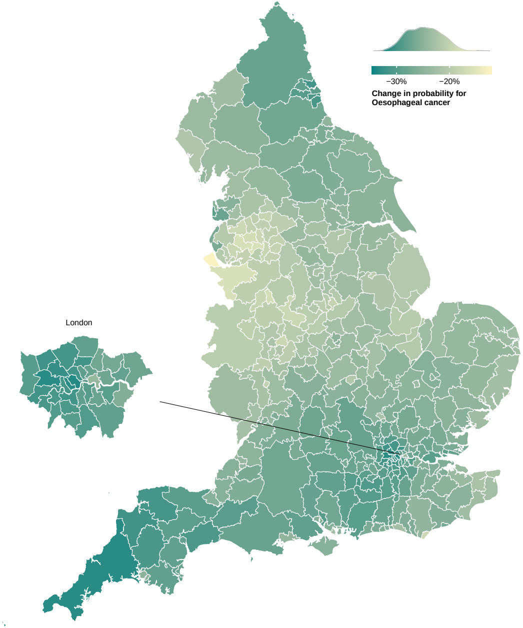

Oesophageal cancer, men

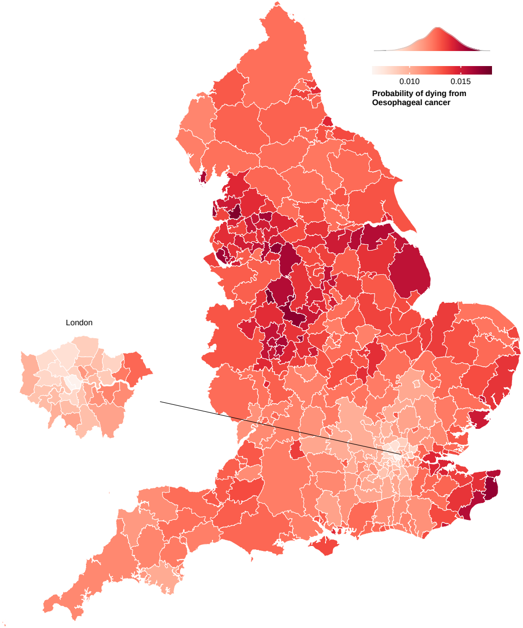

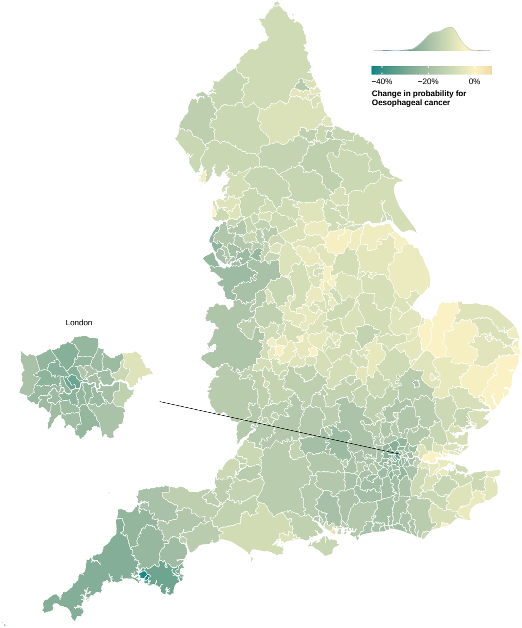

Stomach cancer, women

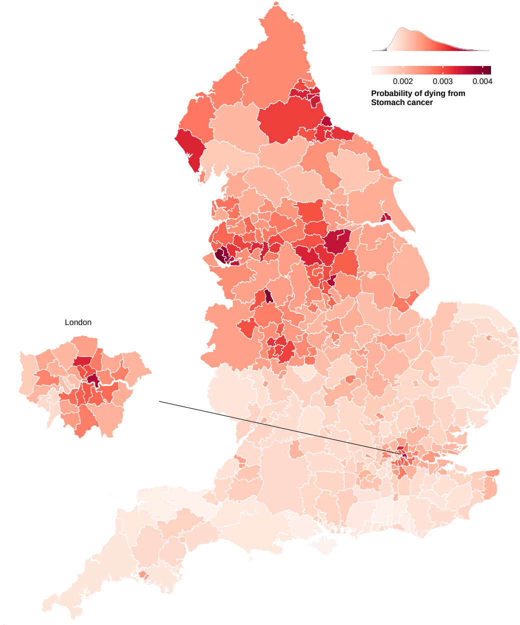

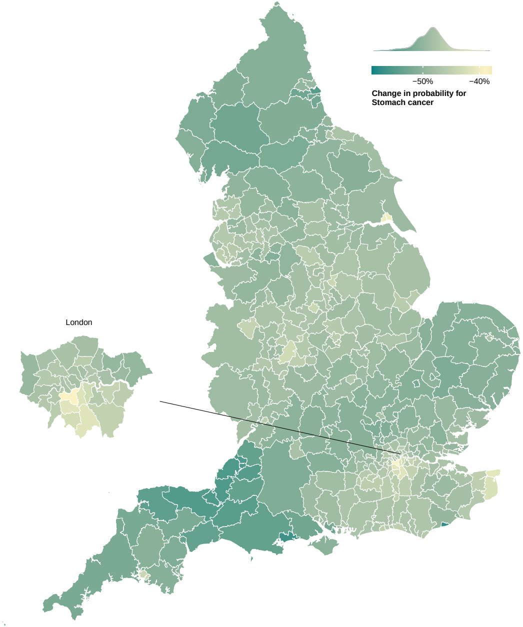

Stomach cancer, men

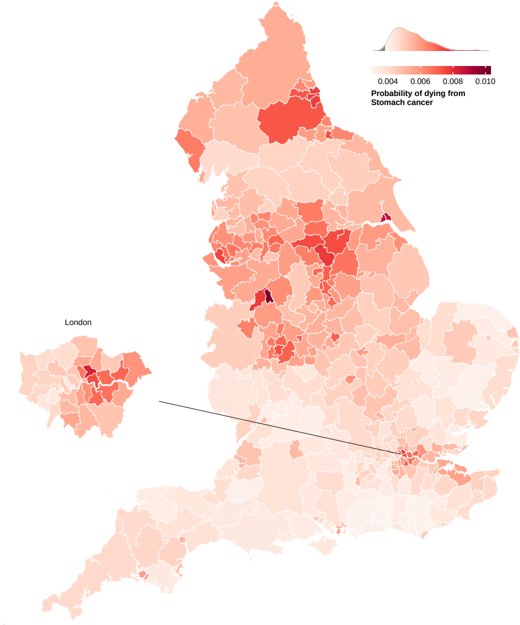

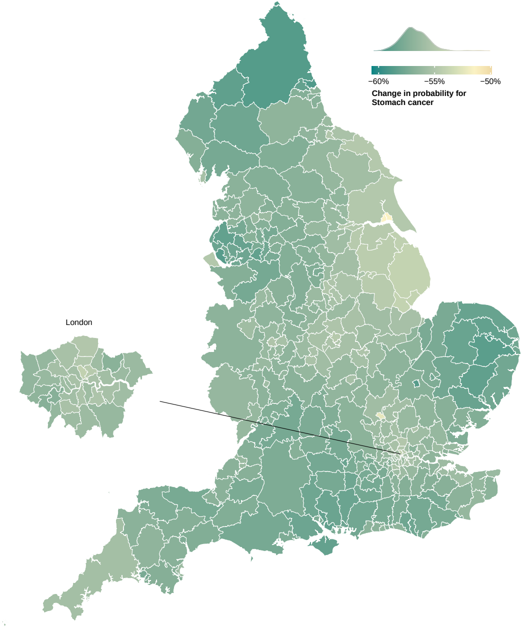

Leukaemia, women

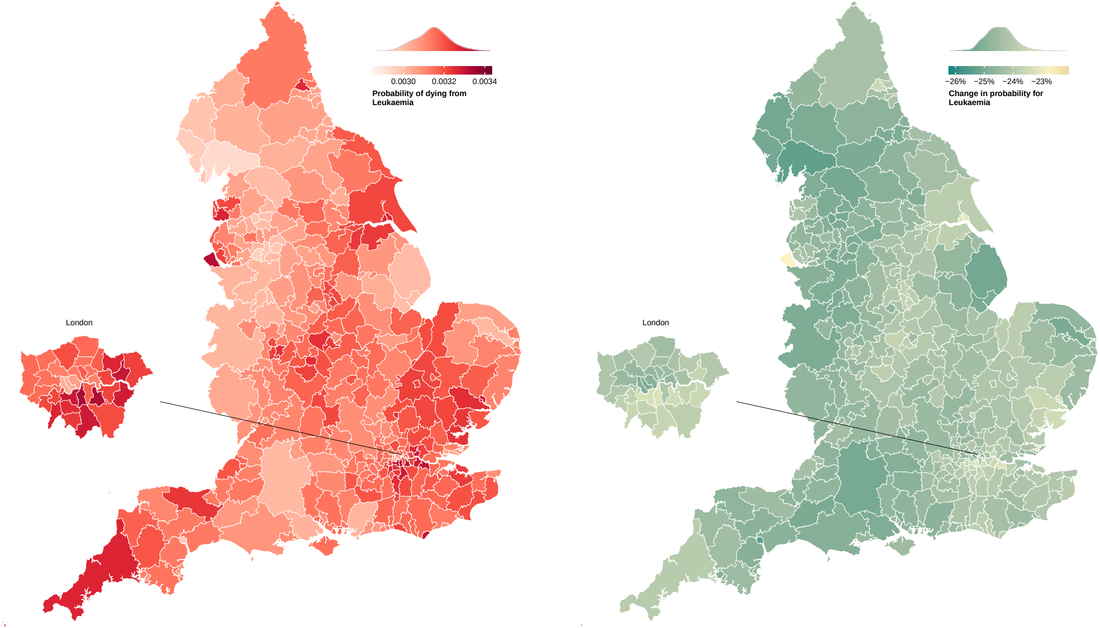

Leukaemia, men

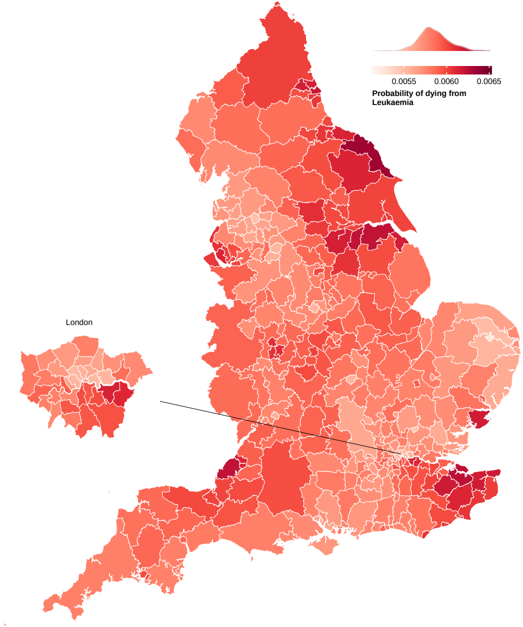

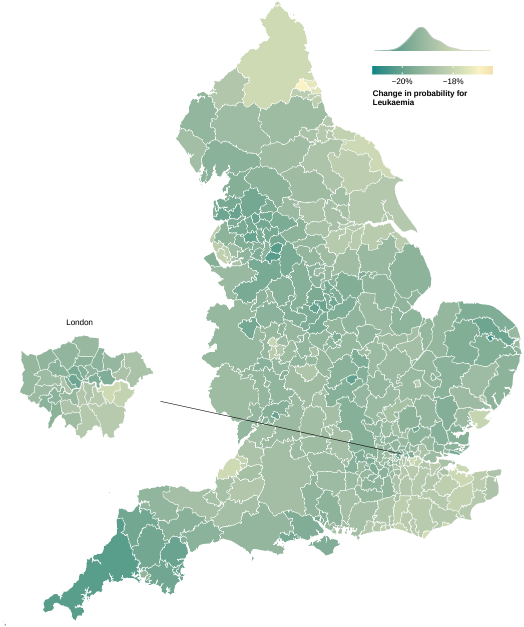

Lymphoma and multiple myeloma, women

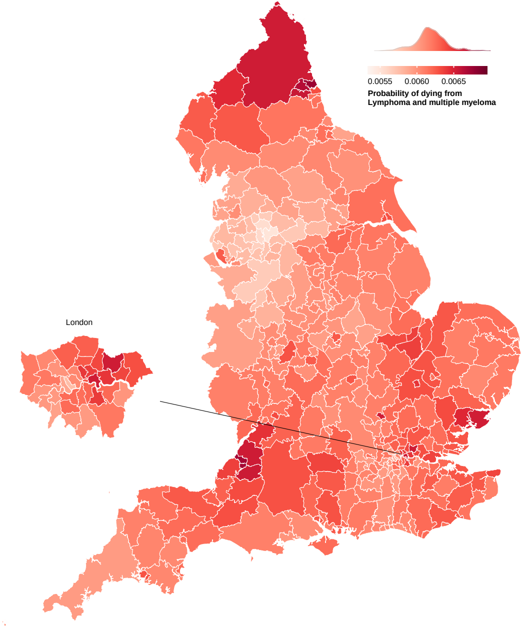

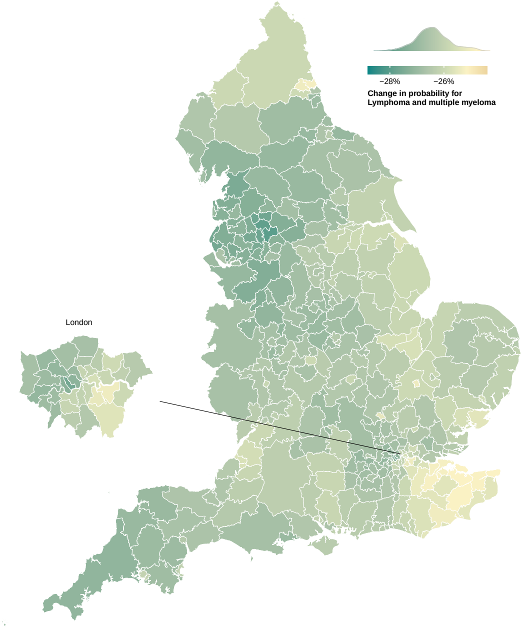

Lymphoma and multiple myeloma, men

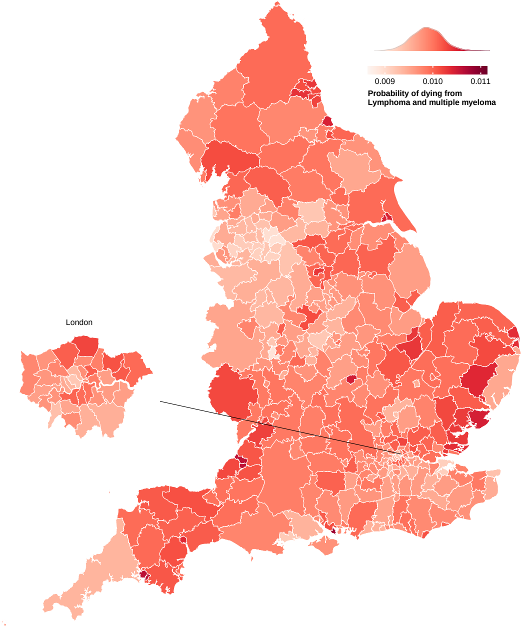

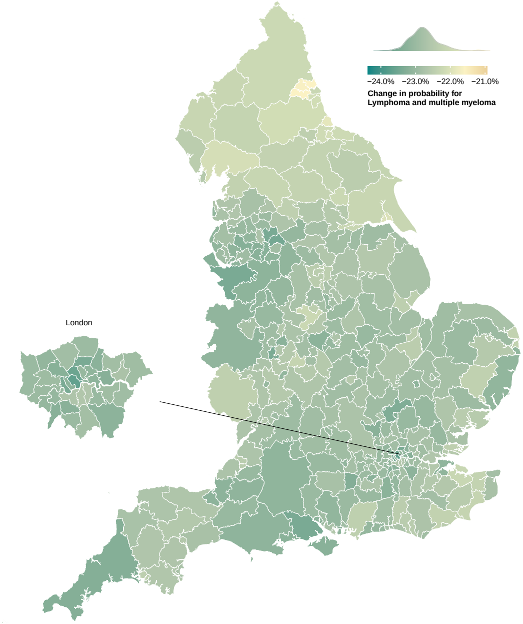

Breast cancer, women

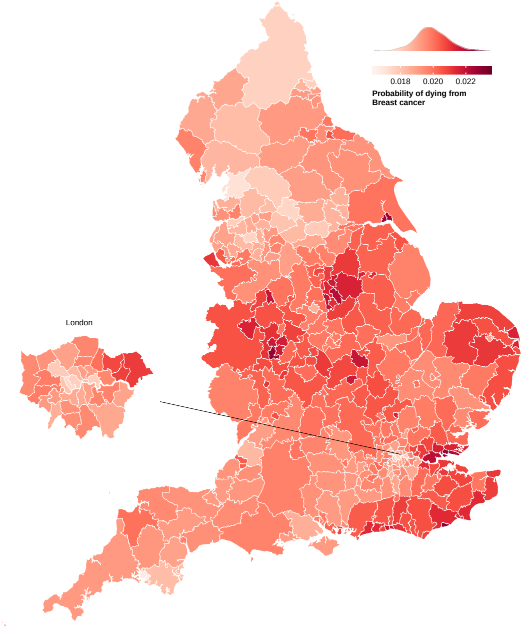

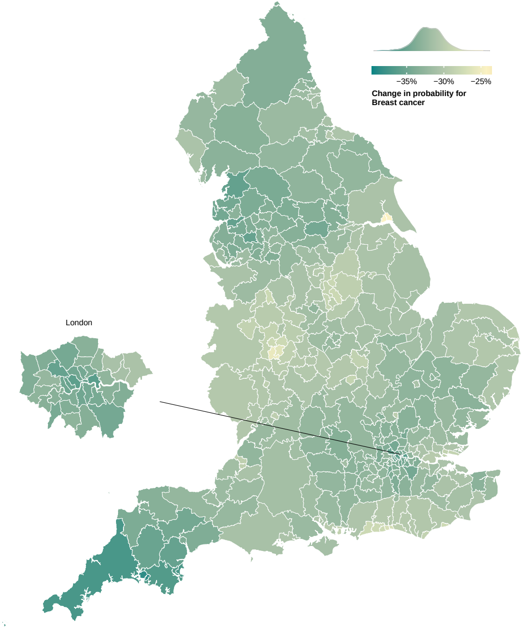

Ovarian cancer, women

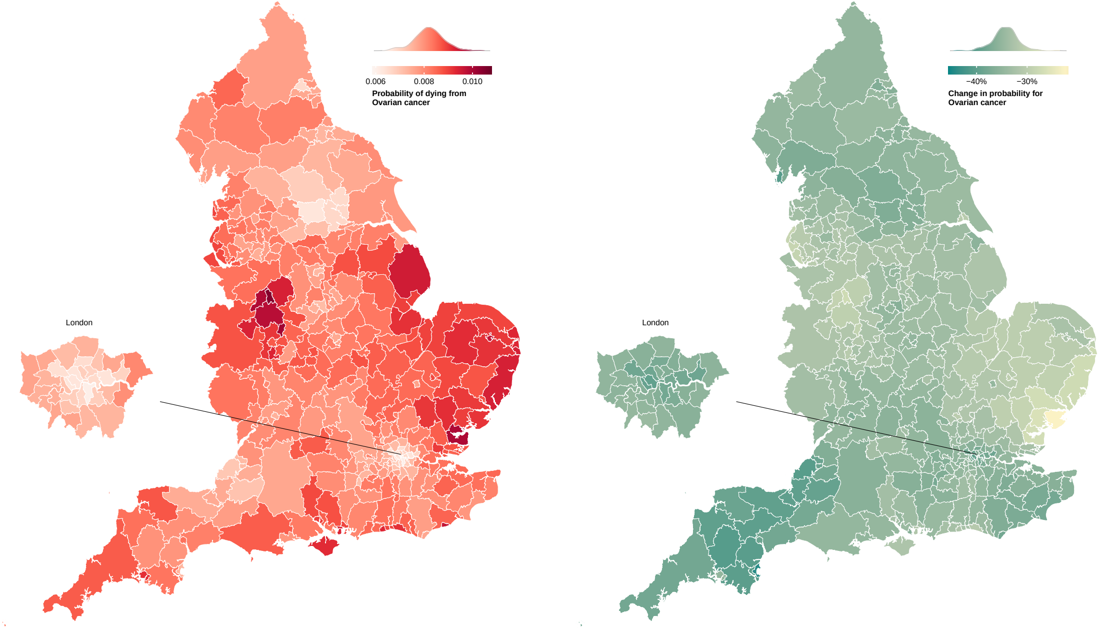

Corpus uteri cancer, women

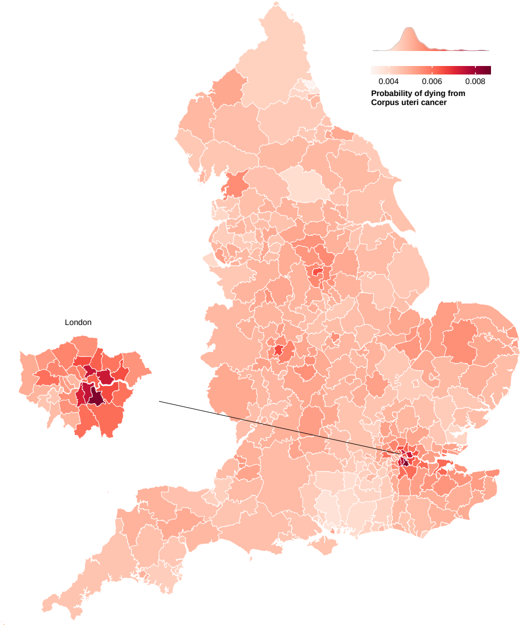

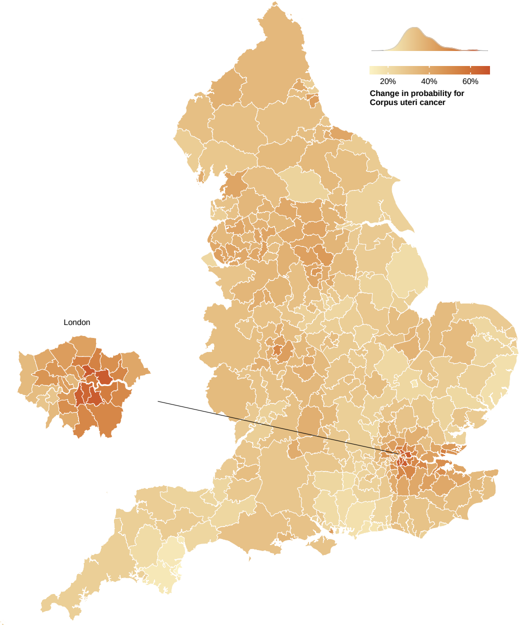

Prostate cancer, men

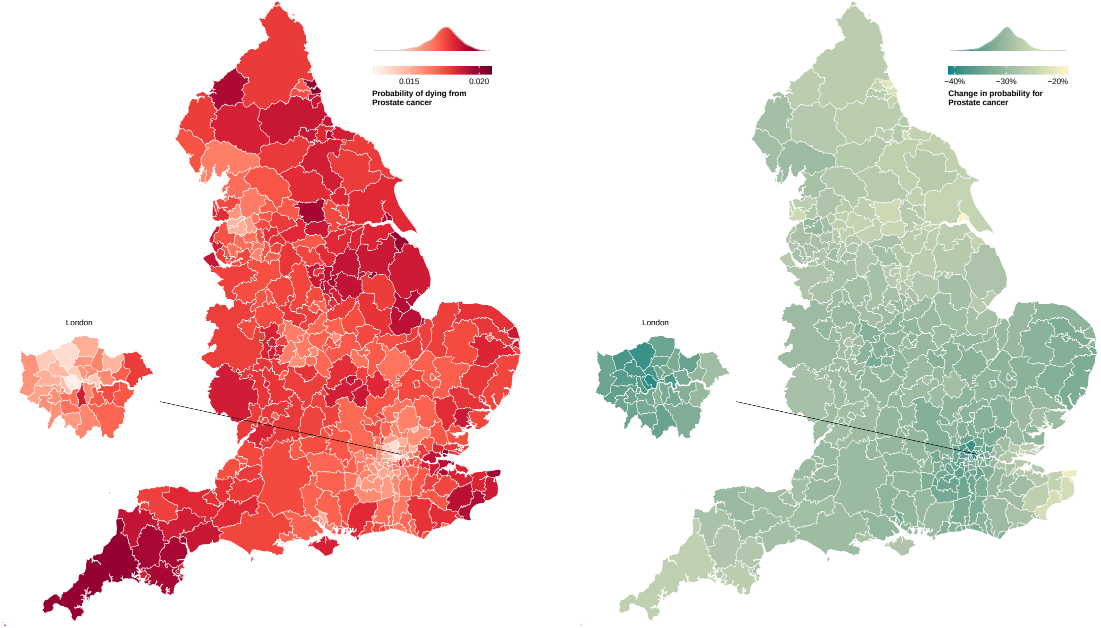

Liver cancer, men

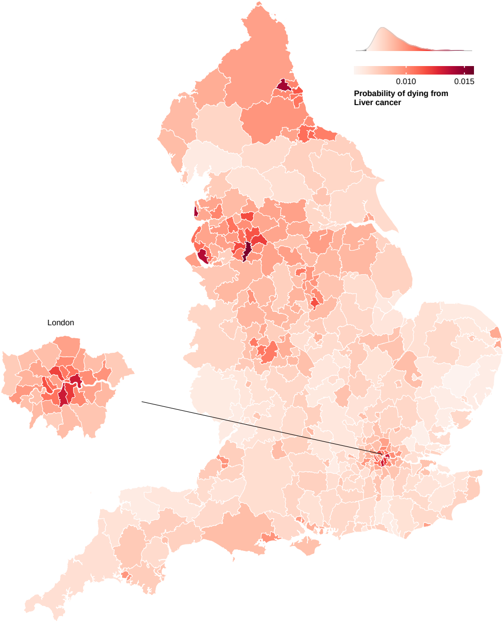

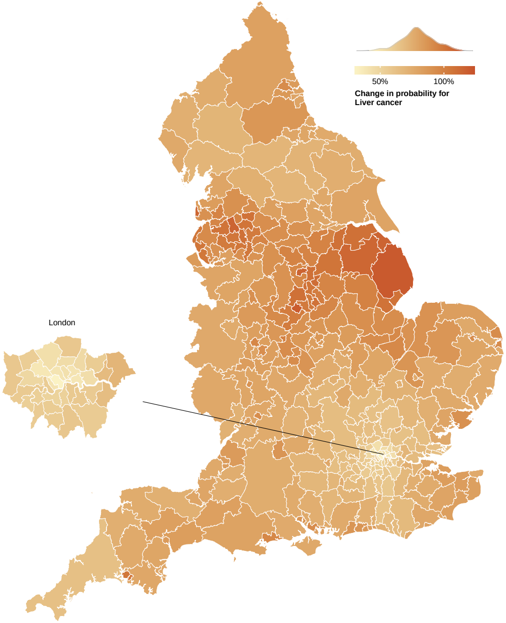

Bladder cancer, men

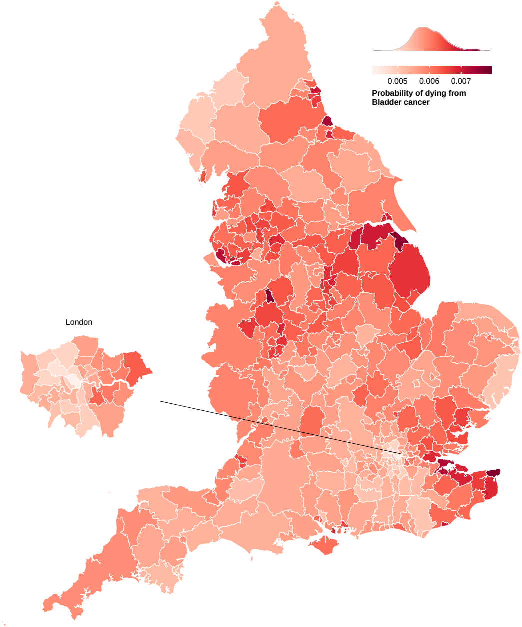

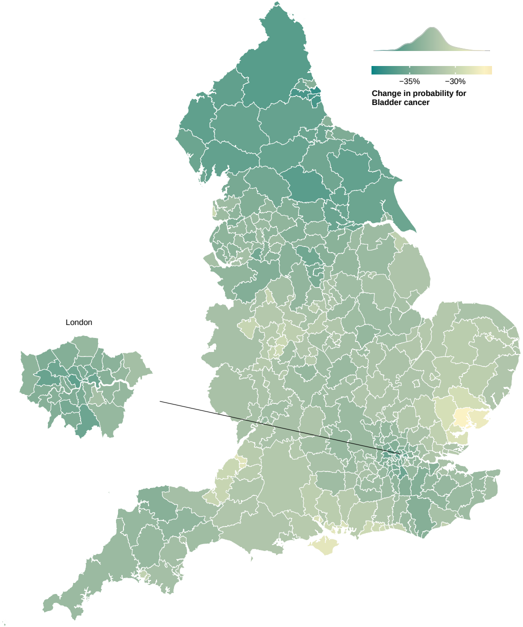

All other cancers, women

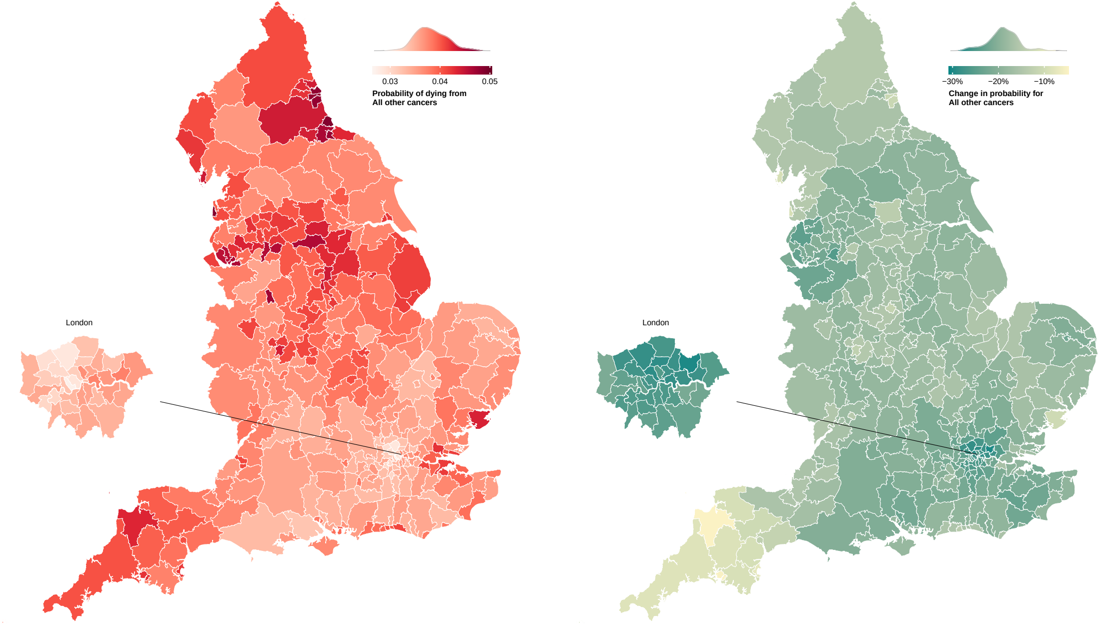

All other cancers, men

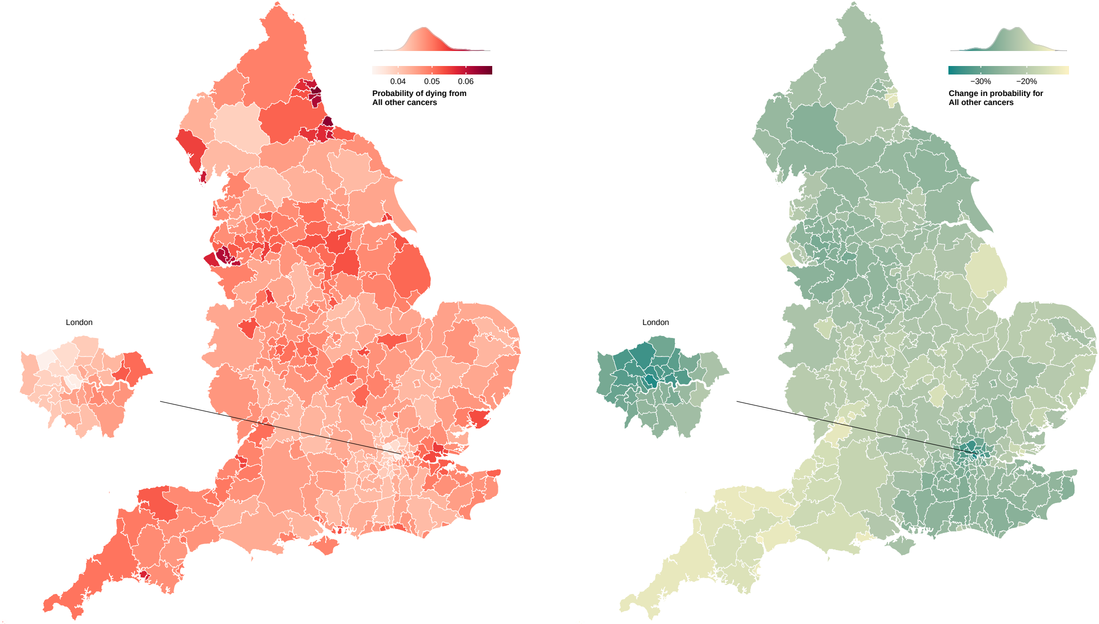

Appendix Figure 3: Pairwise correlations between the leading cancers in 2019, by sex.

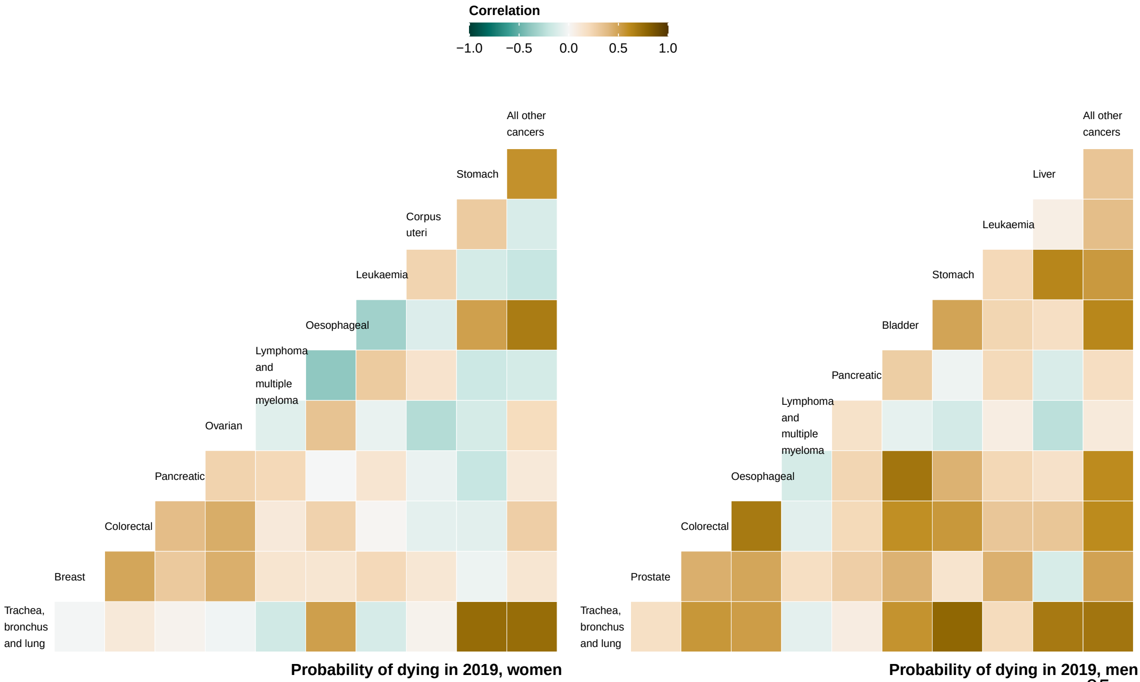
Correlation
−1.0
−0.5
0.0
0.5
1.0
All other cancers
Stomach
Corpus uteri
Leukaemia
Oesophageal
Lymphoma and multiple myeloma
Ovarian
Pancreatic
Colorectal
Breast
Trachea, bronchus and lung
Probability of dying in 2019, women
All other cancers
Liver
Leukaemia
Stomach
Bladder
Pancreatic
Lymphoma and multiple myeloma
Oesophageal
Colorectal
Prostate
Trachea, bronchus and lung
Probability of dying in 2019, men

References

1 Rashid T, Bennett JE, Paciorek CJ, *et al.* Life expectancy and risk of death in 6791 communities in England from 2002 to 2019: high-resolution spatiotemporal analysis of civil registration data. *The Lancet Public Health* 2021; **6**: e805–16.

2 Bennett JE, Rashid T, Zolfaghari A, *et al.* Changes in life expectancy and house prices in London from 2002 to 2019: hyper-resolution spatiotemporal analysis of death registration and real estate data. *The Lancet Regional Health – Europe* 2023; **27**: 100580.

3 Besag J, York J, Mollié A. Bayesian image restoration, with two applications in spatial statistics. *Ann Inst Stat Math* 1991; **43**: 1–20.

4 Kontis V, Bennett JE, Mathers CD, Li G, Foreman K, Ezzati M. Future life expectancy in 35 industrialised countries: projections with a Bayesian model ensemble. *The Lancet* 2017; **389**: 1323–35.

5 Bennett JE, Li G, Foreman K, *et al.* The future of life expectancy and life expectancy inequalities in England and Wales: Bayesian spatiotemporal forecasting. *The Lancet* 2015; **386**: 163–70.

6 Phan D, Pradhan N, Jankowiak M. Composable Effects for Flexible and Accelerated Probabilistic Programming in NumPyro. 2019; published online Dec 24. DOI:10.48550/arXiv.1912.11554.

7 Vehtari A, Gelman A, Simpson D, Carpenter B, Bürkner P-C. Rank-normalization, folding, and localization: An improved R-hat for assessing convergence of MCMC. *Bayesian Anal* 2021; **16**: 667–718.

8 Preston SH, Heuveline P, Guillot M. Demography: Measuring and Modeling Population Processes. Blackwell Publishing, 2001.
